# Study of Population-Differentiated Single-Nucleotide Variants in Human Immunoglobulin Loci Using Short-Read Sequencing Data

**DOI:** 10.3390/genes17101216

**Published:** 2026-09-30

**Authors:** Qi Zhang, Xudong Chen, Qiang Li

**Affiliations:** Translational Medical Center for Development and Disease, Shanghai Key Laboratory of Birth Defect Prevention and Control, NHC Key Laboratory of Neonatal Diseases, Institute of Pediatrics, Children’s Hospital of Fudan University, National Children’s Medical Center, Shanghai 201102, China; qiqi_zhang@fudan.edu.cn (Q.Z.); cardiac_chenxd@163.com (X.C.)

**Keywords:** immunoglobulin loci, population genetics, genetic differentiation, antibody diversity, regulatory annotation

## Abstract

Background/Objectives: Population differences in immunoglobulin (IG) variation offer a starting point for studying inherited antibody diversity. We screened reference-based single-nucleotide variants for population differentiation and linked the resulting candidates to expression and trait annotations. Methods: The primary analysis included 2488 participants from five 1000 Genomes Project groups after pruning documented pedigree relationships and incorporated reference-risk filtering, genotype-quality sensitivity, two FST estimators, linkage disequilibrium and resampling. A separate descriptive extension included 255 Simons Genome Diversity Project participants but did not constitute independent replication. Results: The four-estimate screening rule retained 231 conditional candidates: 49 in IGH and 182 in IGL, with none in IGK. Of these, 177 matched GTEx significant-eQTL catalogs, and seven coordinates overlapped 18 GWAS Catalog records. At rs2856876, the observed alternate-allele frequency was 70.04% in East Asian and 13.65% in European samples; annotations included an IGLV4-69 expression association and a protein-level trait with the same gene label. This correspondence identifies a question for molecular follow-up, not cross-assay validation. High-FST candidates were not consistently more likely to appear in GTEx catalogs than variants in supported local backgrounds. Local assembly–call disagreement, LD dependence and SGDP population composition further constrained interpretation. Conclusions: The resource connects conditional population differences with specific expression and molecular-trait records for follow-up studies of antibody variation.

## 1. Introduction

The allelic diversity of immunoglobulins (IGs) provides an inherited foundation for the human immune system to recognize diverse antigens and contributes to differences in antibody repertoires among individuals. At the genomic level, IG loci contain single-nucleotide variants (SNVs), insertions and deletions, and extensive copy-number and structural variation [1,2,3]. These forms of variation can change the sequence and availability of gene segments used during V(D)J recombination. Studies integrating genomic and repertoire data have demonstrated associations with both naive and antigen-experienced antibody repertoires [2,3]. Allele and gene-copy frequencies also differ among sampled human populations, providing a reason to investigate how inherited IG variation contributes to antibody diversity at the population level [1,4].

Studies of IGHV1-69 illustrate how allele context can influence particular antibody responses. Its germline alleles, copy number and repertoire usage have been associated with anti-influenza responses, and allele substitutions altered the activity of selected SARS-CoV-2-neutralizing antibodies [4,5]. These examples, together with heavy- and light-chain repertoire studies, motivate investigating population variation without assigning a uniform antiviral function to an entire V gene [2,3].

The three major IG loci offer complementary perspectives on this diversity. IGH encodes heavy-chain components involved in antigen recognition and Fc-mediated effector functions, whereas IGK and IGL encode light chains that also contribute directly to the antigen-binding site. Variation at both heavy- and light-chain loci can therefore matter for the antibodies that an individual produces [3,6]. Population differences in their allele frequencies motivate questions about repertoire composition, infection and vaccine responses, and immune-mediated disease.

Global genomic resources, including the 1000 Genomes Project (1KGP) and Simons Genome Diversity Project (SGDP), provide a basis for examining human variation across geographic groups [7,8]. Specialized studies have already established substantial IG diversity and its repertoire consequences [1,2,3]. A complementary question remains: which population-differentiated SNVs in existing reference-based resources coincide with expression or trait associations worth investigating? Describing allele-frequency differences alone cannot answer that question. Integrating population comparisons with antibody-related annotations, the GWAS Catalog and tissue-specific expression data can connect candidate variants to defined biological measurements, provided that annotation overlap is distinguished from functional evidence.

Short-read calls at IG loci require caution because sequence similarity, structural variation and somatic rearrangements in lymphoblastoid cell lines (LCLs) can distort inherited-genotype inference [9,10,11,12]. We therefore evaluate reference and genotype sensitivity while retaining the need for independent germline validation.

In this study, we examined population-differentiated SNVs across IGH, IGK and IGL and used GTEx and GWAS annotations to identify specific questions for antibody-genetics research. The primary analysis used five 1KGP geographic groups, with explicit reference-risk, genotype-quality, estimator, LD and sampling checks. SGDP provided a separate description of the fixed candidates across seven geographic groups, rather than an independent replication cohort or a pooled source of FST estimates. We then examined expression and trait records for the resulting candidates, including their tissue context and the limits of available background comparisons. Our aim was to use population variation to define biologically interpretable follow-up questions, while acknowledging uncertainty in the underlying calls.

## 2. Materials and Methods

### 2.1. Source Material, Cohort Roles and Sample Inclusion

We used individual-level regional VCFs and archived analysis outputs associated with 1KGP and SGDP. The high-coverage 1KGP resource and SGDP study provide context for these data [7,8]. The 1KGP primary panel comprised 2504 identifiers shared by the three regional VCF headers and the established population panel. We applied a deterministic, lexicographically ordered greedy independent-set rule to a graph of explicit blood relationships and shared known parents in the corrected pedigree file dated 4 July 2025. Family identifiers alone did not create edges, and no transitive closure or genotype-quality-based selection was used. Removing 16 known-pedigree redundancies left 2488 participants: AFR, 649; AMR, 347; EAS, 504; EUR, 503; and SAS, 485. These labels denote the project’s broad geographic groupings, not homogeneous biological populations. A further 698 identifiers in the expanded local headers were outside the chosen panel, not excluded for poor quality. We did not use genome-wide kinship estimates to establish the absence of all cryptic relatedness.

**Figure 1:**
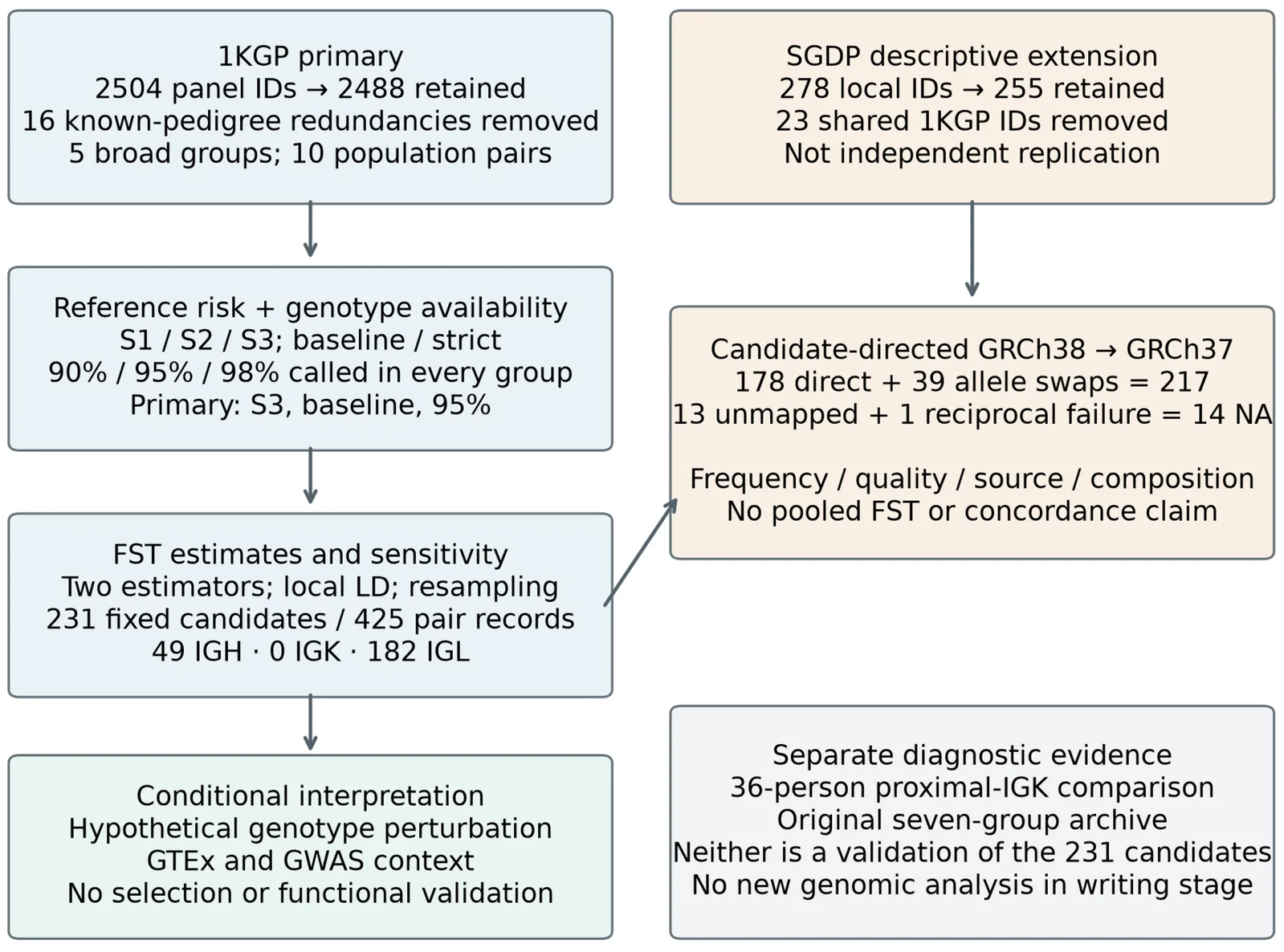
Cohort roles and the reconstructed workflow. The 1KGP branch supplies primary five-group FST summaries and fixed candidates. The SGDP branch describes the harmonized candidates separately, including unavailable correspondences; removal of shared IDs does not establish independence. S1–S3 are reference-risk site sets, not accuracy classes. The local assembly comparison and historical seven-group archive are diagnostic evidence outside the candidate-discovery procedure. All counts refer to their explicitly named branch.

The SGDP local headers contained 278 individuals; removing 23 identifiers shared with 1KGP left 255 participants for the descriptive extension (Table 1). Identifier exclusion prevents direct identifier overlap but does not establish genetic unrelatedness or remove imputation dependence. Figure 1 shows cohort roles and the reconstructed workflow.

### 2.2. Coordinates, Allele Identity and Regional Scope

Reference-risk annotation used GRCh38 BED intervals, with zero-based, half-open coordinates: IGH, chr14:105,586,436–106,879,844; IGK, chr2:88,857,360–90,235,368; and IGL, chr22:22,026,075–22,922,913. Reported variant positions are one-based. The primary 1KGP SNV key was chromosome, position, reference allele and alternate allele.

For the SGDP extension, we mapped fixed GRCh38 candidate keys to the local GRCh37 representation and checked both reference sequences, reciprocal coordinates and allele identity. Direct correspondences were distinguished from reference/alternate swaps. Accepted swaps required the consistent recoding of GT, GP, PL and AD, with the GRCh38 alternate allele retained as the frequency target. Failed or absent correspondences remained unavailable rather than being assigned homozygous-reference genotypes. This candidate-directed procedure differs from the historical full-region GRCh37-to-GRCh38 Picard liftover. The historical full-region Picard operation’s complete success/rejection logs and locus-specific failure causes were not recoverable, so differences between archived before/after counts could not establish liftover failure rates.

To quantify conversion losses in the retained inputs, we performed a separate, newly documented regional audit using Picard LiftoverVcf 2.23.0, the UCSC hg19-to-hg38 chain and an hg38 reference containing all 22 contigs reached by these inputs. All physical records from the three SGDP regional VCFs were projected to sites-only VCFs without genotype or sample columns; duplicate records retained distinct source identities. We compared RECOVER_SWAPPED_REF_ALT = false and true with LIFTOVER_MIN_MATCH = 1.0, strict validation and WARN_ON_MISSING_CONTIG = false. Each input identity was reconciled to exactly one accepted or rejected output per setting, and accepted REF alleles were checked against the target FASTA. Acceptance meant output-file membership, not FILTER = PASS or inclusion in a main IG interval. Reference and chain checksums, commands, per-record outcomes and out-of-region flags are provided in the coordinate-audit deposit described in the Data Availability Statement.

### 2.3. Reference-Risk Strata and Genotype Availability

Reference-risk annotations came from frozen UCSC gap, segmental-duplication, RepeatMasker and simple-repeat tracks and Umap multi-read mappability at 100-mer and 50-mer scales [13]. We assigned five mutually exclusive categories in precedence order: gap/unassessable; mapping/duplication flag; unknown core annotation; repeat only; and no core flag. Supplementary Methods S2 and the packaged code specify the thresholds, versions and hierarchy. A no-core-flag assignment describes the available reference annotation; it does not verify copy assignment, absence of structural variation or intact germline sequence. Individual structural variation and LCL-associated rearrangement were not measured.

S1 comprised simple biallelic SNVs with literal FILTER = PASS. S2 additionally required complete 100-mer core annotations and either repeat-only or no-core-flag status; S3 required no-core-flag status. Complex/multiallelic records were not split into apparently equivalent simple SNVs. Missing, partial, non-diploid and invalid genotypes were unavailable. Baseline filters required GQ ≥20 and DP ≥10; heterozygotes additionally required valid AD and alternate-allele balance between 0.20 and 0.80. Strict filters used GQ ≥ 30, DP ≥ 15 and heterozygous balance between 0.25 and 0.75. Missing or invalid required quality fields failed the relevant filter. Missingness was never filled with reference genotypes. All-five-group call-rate thresholds of 90%, 95% and 98% were evaluated separately.

In practice, these strata determine which sites enter downstream analyses; genotype quality, call rate and candidate eligibility are then assessed separately. For example, the PASS biallelic SNV chr14:105,605,402 C>T (GRCh38) had complete 100-mer core annotations and no core flag, so it was retained in S1, S2 and S3 and subsequently met the candidate rule. A repeat-only site can enter S2 but not S3, whereas a mapping/duplication-flagged site is excluded from both S2 and S3. Retention is not validation of the underlying genotype.

### 2.4. Existing Proximal-IGK Assembly Comparison

We compared existing local short-read calls with paired assembly-derived sequence in 36 overlapping participants across proximal IGK, chr2:88,880,000–89,260,000 (one-based, closed). This region does not cover all IGK and provides no corresponding IGH or IGL benchmark. Simple-coordinate comparisons excluded complex sequence spans and required evaluable sequence in the assembly-derived representation. Both VCF-first and assembly-first views were retained, distinguishing concordant calls, different non-reference genotypes, VCF non-reference/assembly reference, assembly non-reference/VCF reference, absent VCF records and unusable genotypes. A non-reference comparison opportunity was a paired evaluable sample–position with a non-reference genotype in at least one source. An absent VCF record was not assumed to be a reference call.

We compared raw PASS, baseline, strict and baseline-with-50-bp-boundary-padding settings and then added reference-risk annotations to assess disagreement alongside the loss of concordant non-reference opportunities. Neither assembly copy assignment nor the complete original read-to-call history had been independently established. Discrepancies therefore could not be attributed exclusively to short reads or used to estimate true false-positive/false-negative rates, and this comparison did not validate the later candidate set.

### 2.5. FST Estimation, Missingness and Sensitivity Analyses

We calculated Weir–Cockerham and Hudson FST from retained genotype counts, using ratios of summed estimator components for locus-level summaries rather than averages of site-wise ratios [14,15]. The primary settings were S3, baseline genotype quality and ≥95% called participants in each of the five groups, with at least 20 called individuals per group. All combinations of three site sets, two genotype-quality stages and three call-rate thresholds formed 18 settings. Negative estimates were retained. Joint fixation for the same allele gave a non-estimable zero-denominator comparison, whereas opposite fixed alleles were estimable. Filter failure, estimator non-estimability and a negative numerical estimate were kept distinct in the supplied tables; tiny floating-point values were not interpreted as biological differences from zero.

Sensitivity analyses compared quality stages on common estimable positions, restored the 16 excluded pedigree-linked samples on a fixed selected-site set, restricted the maximum all-five-group call-rate spread, and applied local LD pruning. The latter used ascending-position greedy retention within 50 kb and removed a later site when pairwise-complete dosage r^2^ exceeded 0.2 in any group with adequate information. Each evaluation required ≥20 jointly called individuals, joint minor-allele count ≥ 5 and non-zero variance. Comparisons that could not be evaluated remained unassessed rather than being assigned r^2^ = 0; the retained set was not certified to be globally independent.

For locus summaries, spatial resampling used fixed chromosome-anchored 50 kb and 100 kb blocks, 1000 replicates. Percentile intervals required ≥10 informative blocks and ≥950 valid draws. These are conditional spatial intervals, not genotype-error intervals. Separately, 100 balanced downsampling replicates retained 200 individuals per broad group, using proportional quotas for the 26 original population strata, sampling without replacement. The full-sample primary site mask remained fixed, without reapplying the 95% threshold. This distribution assesses sample-size sensitivity, not a confidence interval.

### 2.6. Conditional Candidates and Individual-Resampling Intervals

The exploratory candidate rule was fixed after inspection of aggregate FST sensitivity results and before generating the candidate lists; it was not preregistered before the study. A site–population-pair comparison had to satisfy S3 and 95% call rates in both quality stages, finite positive denominators for both estimators, all-five-group call-rate spread ≤ 0.02 in each stage, and pair-pooled minor-allele count ≥ 20 in each stage. A high comparison required all four estimates to exceed 0.25 + 10^−10^ and the alternate-allele-frequency difference to have the same non-zero direction in both quality stages. Low comparisons required all four estimates below 0.05 − 10^−10^, without a direction requirement. Inclusive guard bands around either threshold were classified separately before high/low assignment. Thresholds of 0.15 and 0.35 and removal of the minor-allele-count restriction were sensitivity settings. Threshold agreement does not imply close numerical agreement, statistical significance or LD independence.

For the fixed candidates, we resampled genotype-count weights within the 26 original 1KGP strata, preserving each stratum’s sample size. We used 2000 replicates and the seed in the frozen uncertainty configuration, with the same participant weights across variants and quality stages. Percentile 95% intervals required ≥1900 valid replicates. These intervals condition on the observed genotypes, chosen strata and selected candidates; they are neither selection-adjusted nor simultaneous and exclude sequencing, reference, SV and LCL uncertainty. Empirically degenerate intervals were explicitly marked.

### 2.7. Hypothetical Genotype Perturbation and Reference-Risk Background

We evaluated eight deterministic scenarios on the fixed candidate–pair set, at replacement-opportunity fractions from 0 to 20% in increments of 0.1 percentage points. Within each pair, baseline alternate-allele frequency defined the high- and low-frequency groups. Scenarios replaced accepted genotypes toward reference or alternate homozygotes in one or both groups, including convergent and divergent frequency perturbations. Expected genotype counts were inserted into the FST formulas: for replacement opportunity e and target homozygote t∈{0, 1}, AC′ = (1 − e)AC + 2ent and H′ = (1 − e)H, with called n unchanged. This is FST evaluated at expected counts, not E(FST) under a stochastic experiment. An opportunity rate is not the fraction of genotypes that actually change, and neither quantity is an empirically measured error rate. Each pair defines its own hypothetical scenario; these are not a globally consistent perturbed callset. No power or false-positive/false-negative count was inferred from them.

A separate analysis assessed reference-risk distributions before applying S2/S3 exclusions. Within each single estimator/quality-stage S1 setting with all-five-group call rates ≥ 95%, independently estimable values >0.25 + 10^−10^ or <0.05 − 10^−10^ defined high or low sites, respectively; these are not the four-estimate fixed-candidate classes of Section 2.6. Sites were compared only in shared strata defined by locus, population pair, chromosome-anchored 100 kb block, pair-pooled minor-allele-frequency bin and minimum all-five-group call-rate bin. Low-site category frequencies were standardized to supported high-site counts. The complete five-category 100-mer distribution was the primary endpoint, and 50-mer categories and alternative settings were sensitivities. Unsupported high sites and comparisons without shared strata were reported.

### 2.8. SGDP Frequency, Source and Composition Sensitivity

SGDP summaries were restricted to harmonized fixed candidates under three genotype scenarios: parseable GT; GT with called-genotype GP ≥ 0.90; and the latter GP filter plus total AD ≥ 8 and at least two reads for each called allele. PL was recoded during allele swaps but was not used as the quality threshold. A VCF FILTER value of “.” indicates that filters were not applied and was not treated as “PASS”. Cell-line, blood and saliva metadata labels were retained as reported; a cell-line label did not establish participant-level EBV-LCL provenance.

We computed alternate-allele counts, called chromosome denominators, missingness and individual-weighted regional frequencies, alongside equal-Population-ID weighting and leave-one-Population-ID-out summaries. Conditional percentile intervals used 1000 within-Population-ID resamples, fixed group sizes, ≥10 called individuals, call rate ≥ 0.90 and ≥950 valid replicates. These intervals can be degenerate and do not capture between-population sampling or imputation uncertainty. Unsupported source strata remained unsupported. Absolute shifts ≥ 0.05 were descriptive flags, not hypothesis tests or exclusion criteria. No individuals were excluded on the basis of whether their removal increased or decreased a frequency. Source-stratified SGDP descriptions were not substituted for the requested matched 1KGP LCL/non-LCL comparison. Precise equal-Population-ID frequencies and complete fine-group or nested frequency tables are withheld from the public presentation to limit inference about individual samples in very small strata. Aggregate sensitivity counts and selected regional summaries are retained; the underlying analyses and sample inclusion are unchanged.

### 2.9. GTEx and GWAS Annotation

GTEx v11 significant cis-eQTL records were examined for whole blood, spleen, EBV-transformed lymphocytes and terminal ileum, using an exact chromosome–position–reference–alternate GRCh38 key [16]. We retained tissue, gene, slope, source nominal *p*-value, source gene-level q-value and whether a candidate was the listed gene–tissue lead variant.

For an IG-local background description, high and low candidate-rule comparisons were restricted to shared strata defined by locus, pair, 100 kb block, baseline pair-pooled minor-allele-frequency bin and minimum call rate across both stages and all five groups. Low catalog-presence rates were standardized to supported high counts; equal-cell weighting and the subset retained by the preceding LD analysis were sensitivity analyses.

We queried the GWAS Catalog association records (download dated 4 September 2026) for single-coordinate matches [17]. Coordinate overlap, current RefSNP membership and original-study effect-allele identity were recorded separately.

### 2.10. Analytical Scope and Unresolved Measurement Limitations

The analyses describe sensitivity within archived reference-based calls. The proximal-IGK comparison measures disagreement between specific inputs, not an unbiased proportion of unreliable calls across IGH, IGK and IGL. No verified, adequately comparable 1KGP non-LCL control group, individual SV reconstruction or independent validation of the selected high-FST candidates was available. Reference filtering and candidate-directed coordinate checks cannot recover missing haplotypes or eliminate population-dependent ascertainment. Incomplete source histories, SGDP imputation dependence and observed sampling composition further restrict generalizability. Within these limits, filtering, resampling and hypothetical perturbations assess different aspects of sensitivity.

Analyses used custom scripts in Python 3.12.14 with NumPy 2.3.5; the reference-risk annotation module used Python 3.12.9, pysam 0.23.3 and HTSlib 1.21. GTEx annotation and background comparisons used PyArrow 20.0.0, and native LD code was compiled with Apple clang 21.0.0. Picard LiftoverVcf 2.23.0 was used for the coordinate-conversion audit. Module-specific software records, parameters and source versions are provided in the Supplementary Methods and accompanying provenance tables. This computational study used existing data and involved no new experimental devices or commercial reagents.

## 3. Results

### 3.1. The Analysis Covered Three IG Loci with Unequal Reference and Genotype Support

The primary analysis included 2488 known-pedigree-pruned 1KGP participants and 10 pairwise comparisons across five geographic groups, and 255 SGDP participants supplied a separate descriptive extension (Table 1). Reference filtering restricted the genomic space differently across loci. Mapping/duplication flags covered 55.86% of IGH, 67.42% of IGK and 27.92% of IGL, while no-core-flag sequence covered 19.36%, 4.49% and 38.61%, respectively. IGK also contained 268,000 reference-gap bases, or 19.45% of its interval. These are reference-annotation proportions, not genotype-error rates.

From 70,344 IGH, 56,134 IGK and 43,044 IGL S1 sites, S3 reference filtering retained 15,582, 3533 and 17,141. Baseline genotype filtering and ≥95% call rates in all five groups left 5843, 1273 and 12,276 selected positions (Table 2). Pair-specific estimator eligibility further restricted the primary comparisons to 2081–3249 IGH, 449–696 IGK and 4739–7329 IGL positions. The candidate survey therefore describes a restricted and unequally represented part of each locus.

A site that varies across the five-group panel can nevertheless be fixed for the same allele in a particular population pair, giving a zero FST denominator despite passing quality filters. Across the ten primary S3/baseline/95% comparisons, this accounted for 32,879/58,430 IGH (56.27%), 7029/12,730 IGK (55.22%) and 64,094/122,760 IGL (52.21%) site–pair records; no other non-estimability was recorded in this setting (Supplementary Data S3). These post-filter counts describe shared fixation in the accepted calls, not technical filter failures or the unresolved causes of historical NA entries.

### 3.2. Conditional Candidates Identified Population Differences in IGH and IGL

**Figure 2:**
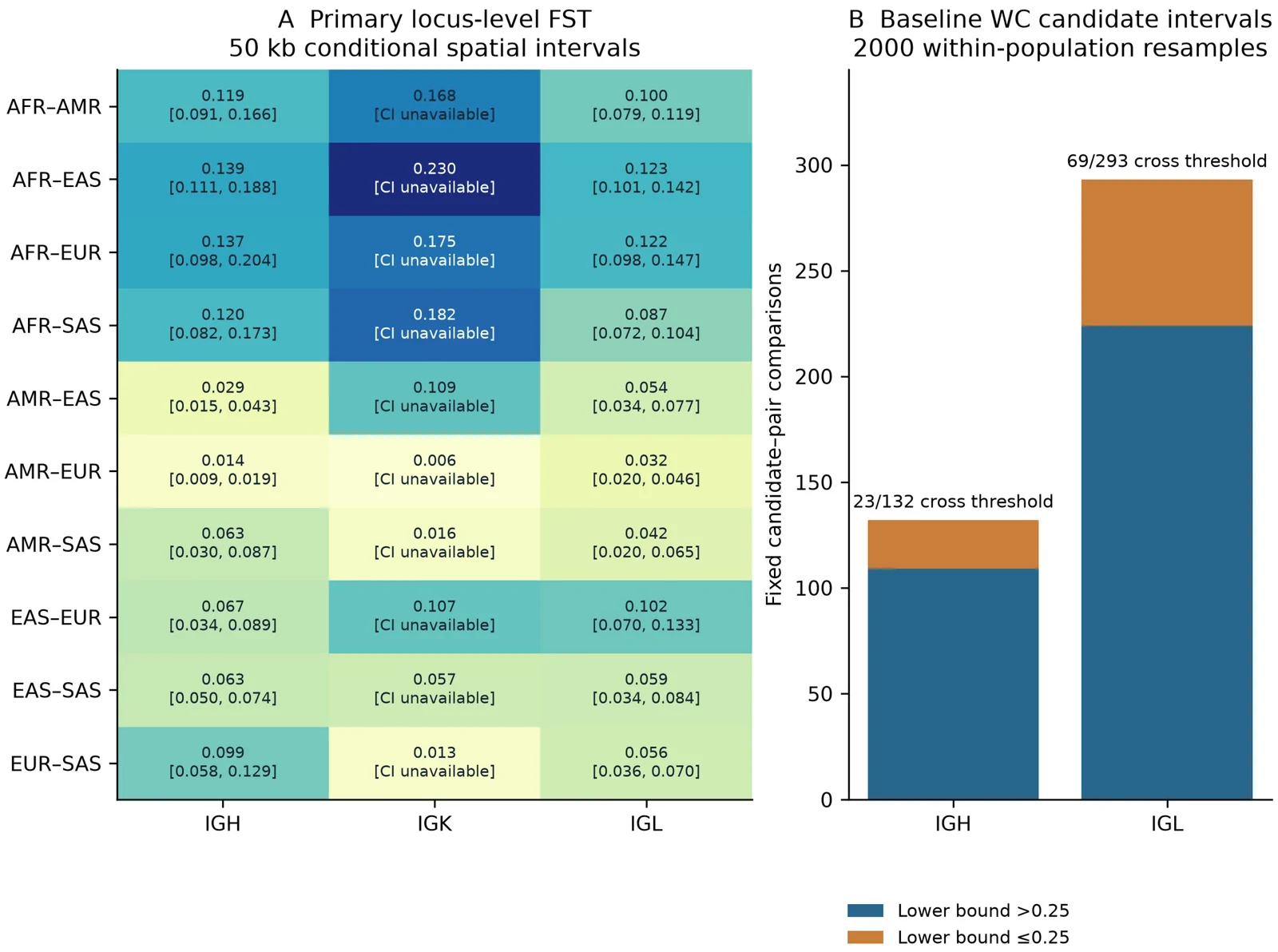
Primary FST estimates and conditional interval sensitivity. (**A**) Weir–Cockerham ratios of component sums for S3/baseline/95% across all 10 population pairs. Brackets show 95% conditional 50 kb spatial bootstrap intervals (1000 replicates) where ≥10 informative blocks were present; IGK intervals are unavailable, not zero. The color scale runs from pale yellow (lower estimates) to dark blue (higher estimates), spans 0–0.25 and does not indicate significance. Complete signed estimates, estimator variants and denominator/status fields are in Supplementary Data S3. (**B**) Numbers of fixed candidate–pair baseline Weir–Cockerham intervals with lower bounds above or at/below 0.25, from 2000 within-population individual-resampling replicates. Blue denotes lower bounds above 0.25, and orange denotes lower bounds at or below 0.25. These candidate intervals differ from panel A’s spatial intervals and are not selection-adjusted, simultaneous or genotype-error intervals. All 425 candidate–pair records are represented; IGK has no candidates under this rule.

The rule requiring high FST in both estimators at both genotype-quality stages retained 231 unique candidates: 49 in IGH and 182 in IGL, with none in IGK. They contributed 425 high candidate–pair comparisons, comprising 132 in IGH and 293 in IGL (Figure 2; Supplementary Data S4). This is agreement in a threshold category, not a count of independent genetic signals: only three IGH and eight IGL candidates belonged to the inherited locally LD-retained set. The absence of IGK candidates under this rule does not establish biological conservation.

The IGL candidate rs2856876 (chr22:22,907,270 C>A) illustrates how the catalog records population differences at a specific allele. Under baseline filters, the observed A frequencies were 706/1008 chromosomes in EAS (70.04%; 504 called participants), 350/1298 in AFR (26.96%; 649), 137/1004 in EUR (13.65%; 502) and 290/966 in SAS (30.02%; 483). AFR–EAS, EAS–EUR and EAS–SAS met the high rule; the four EAS–EUR estimates ranged from 0.49188 to 0.49250. These frequencies apply to the sampled groups and accepted calls. This candidate was removed by the local LD procedure and cannot be treated as an independently supported signal on this evidence; its expression and trait annotations are examined below.

Uncertainty was appreciable even among selected candidates. The baseline Weir–Cockerham interval lower bound was ≤0.25 for 23/132 IGH and 69/293 IGL candidate–pair comparisons. All 1700 FST intervals for the individual estimator/stage combinations met the reporting criteria, while 66 of 2310 allele-frequency intervals were empirically degenerate. These conditional resampling intervals do not include genotyping error or adjust for candidate selection (Supplementary Data S5).

### 3.3. Expression Annotations Connected Candidates to Defined IG Gene and Tissue Contexts

**Figure 3:**
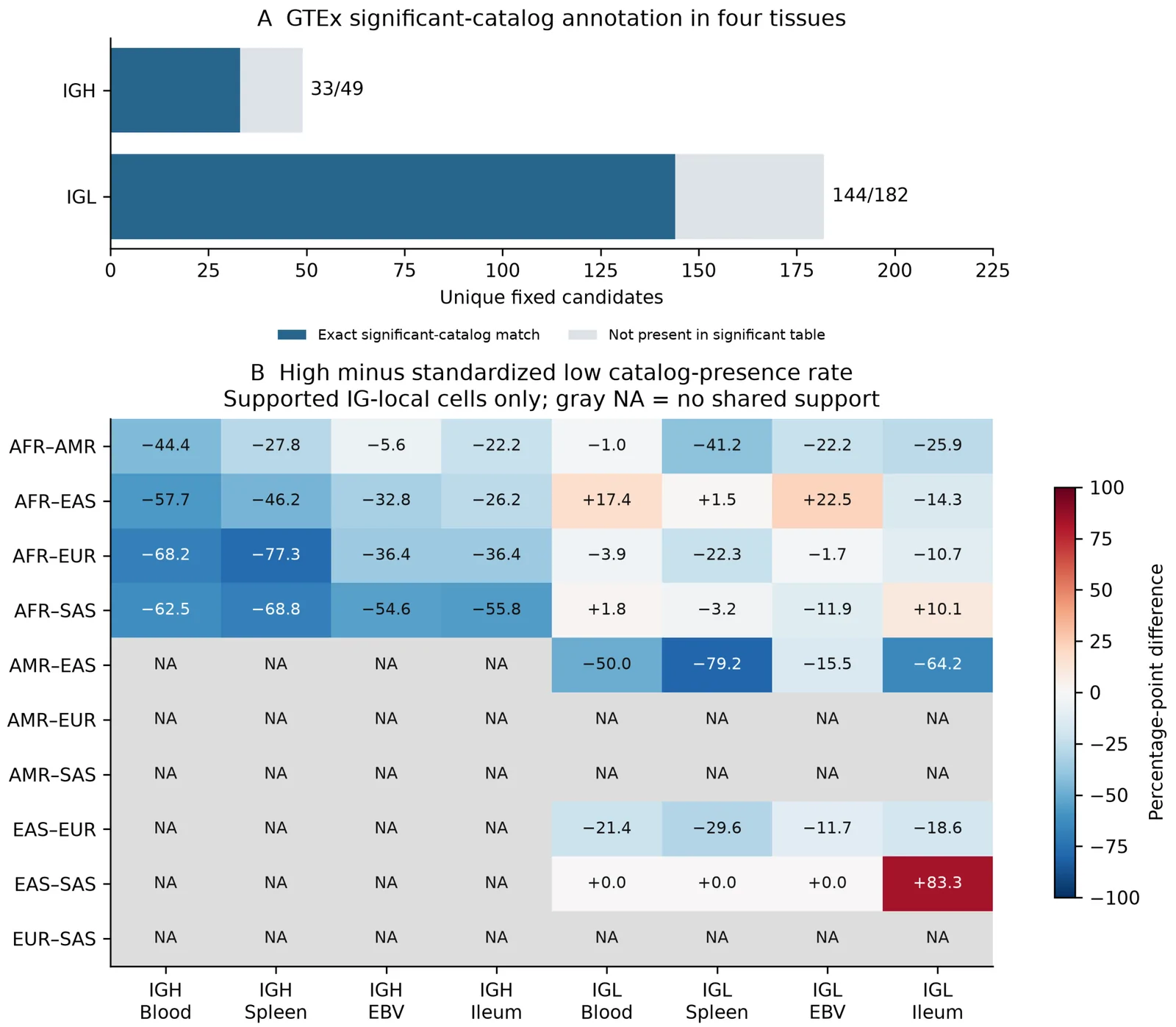
GTEx annotation and support-limited background context. (**A**) Unique fixed candidates with an exact GRCh38 allele match in at least one of four significant-eQTL catalogs. Lack of a match is not a tested negative. IGK is omitted because it has no fixed candidates, not because it lacks regulatory activity. (**B**) All 10 pair-by-four-tissue contrasts in each of IGH and IGL under the all-fixed comparison setting; labels are high minus high-weight-standardized low significant-catalog-presence rates in percentage points. Shared strata match locus, pair, 100 kb block, study-cohort MAF bin and call-rate bin. Gray NA denotes no shared support, distinct from a numerical zero or negative contrast. Exact supported high/all high, supported low and cell counts are in Supplementary Data S7 and the figure-data table. No enrichment *p*-values, cell-composition correction or causal regulatory claims are implied.

GTEx significant-eQTL catalogs matched 177/231 candidates by exact GRCh38 allele identity: 33/49 in IGH and 144/182 in IGL. The matches comprised 1060 variant–gene–tissue records and 92 gene–tissue contexts. IGH candidates mapped to 16 genes in 519 records and IGL candidates to 27 genes in 541 records (Figure 3A; Supplementary Data S6). These counts describe overlapping records, not independent regulatory effects; an unmatched candidate was not necessarily tested or inactive in GTEx. The expression targets included IGLV4-69, IGLV8-61, IGLV9-49 and IGLV7-46, alongside other IG and non-IG transcripts (Supplementary Data S6).

The two source-listed gene–tissue lead records both involved rs2856876. One associated the candidate with IGLV4-69 expression in whole blood (source nominal *p* = 3.93 × 10^−10^; slope = +0.21148); the other associated it with IGLC5 expression in the spleen (*p* = 3.45 × 10^−9^; slope = −0.49297). These concern different genes, not opposite effects on the same gene across tissues. The source annotates IGLC5 as an IG_C_pseudogene, so its transcript association cannot be interpreted as altered production of a functional antibody constant-region protein. Lead status identifies a source-catalog statistic, not a fine-mapped causal variant.

IGHV1-69D provided a different expression context. Twenty-one candidates matched its whole-blood records, and there were 19 matched records in each of EBV-transformed lymphocytes, terminal ileum and spleen; these tissue sets can overlap. None of the 21 whole-blood candidates was the listed lead variant or belonged to the inherited LD-retained set. These associations may tag related regional variation rather than separate regulatory mechanisms. The established antibody literature on IGHV1-69 does not, by gene-name similarity, establish antiviral effects for IGHV1-69D or these candidates.

In supported IG-local background comparisons, high-FST candidates did not consistently have higher catalog-presence rates. All 16 supported IGH pair–tissue contrasts were negative; of 28 IGL contrasts, six were positive, three zero and 19 negative (Figure 3B). Four IGL signs changed under equal-cell weighting. Across all settings, 72/240 comparison units had support, and the locally LD-retained subset supplied only one-to-three supported high comparisons per pair. Thus, the expression matches identify specific associations to examine but do not demonstrate a general excess of regulatory function among high-FST candidates (Supplementary Data S7).

### 3.4. GWAS Overlaps Identified Molecular Traits and an Antibody-Reactivity Measurement

Seven of the 231 candidate coordinates matched 18 GWAS Catalog records with 16 distinct original trait labels: two coordinates were in IGH and five in IGL (Table 3; Supplementary Data S8). The traits mainly concerned quantitative protein measurements and seropositivity, not 16 diseases. All seven candidates had confirmed GRCh38 primary-sequence RefSNP membership, but the allele tested in each original study and its effect direction remained unestablished; four RefSNP records were multiallelic. None of the seven candidates belonged to the inherited locally LD-retained set.

Ten records mapped to rs2856876. Five carried protein-level labels for IGLV5-45, IGLV8-61, IGLV4-69, IGLV7-43 and IGLC2. IGLV8-61 were also among the GTEx expression targets (Supplementary Data S6). IGLV4-69 appears in both the whole-blood expression association and a protein-level trait label, making their relationship a specific question for follow-up. It does not establish that both assays measure the same molecular entity: original-study assay specificity and transcript-to-protein correspondence have not been verified. Nor can the unharmonized GWAS effect allele be joined to the observed A-frequency difference to infer a direction from genotype to expression, protein abundance or immune function. SGDP also flagged this candidate for region-specific sensitivity in America and Central Asia/Siberia.

The IGH candidate rs8009594 (chr14:106,777,494 G>T) overlapped the catalog label “Seropositivity for ruminococcus torques (agilent_118810)” (source *p* = 2 × 10^−8^). The cited study profiled antibody reactivity to peptides using phage-displayed immunoprecipitation sequencing [18]. This places the overlap in an antibody-reactivity context, but the exact peptide-level classification and original effect-allele relationship have not been independently reconstructed here. The record cannot establish infection, microbial abundance or protection; the candidate was LD-removed and had an Oceania sensitivity flag in SGDP.

### 3.5. SGDP Extended the Geographic Description with Composition-Sensitive Frequencies

Of the 231 fixed candidates, 217 could be described in SGDP: 178 direct allele correspondences and 39 reference/alternate swaps, comprising 49 IGH and 168 IGL candidates. Thirteen candidates were unmapped, and one failed the reciprocal check; all 14 remained unavailable. The 255 participants had source labels of cell line (166), blood (74) or saliva (15), not verified participant-level LCL classifications. Across 1519 candidate–region summaries, the largest absolute raw-to-GP-plus-AD frequency shift was 0.0248, and none reached 0.05. This result concerns the available quality fields and does not establish independent genotype accuracy.

The new forward-conversion audit agreed with the expected GRCh38 chromosome, position, REF and ALT for all 217 eligible candidates when swapped-reference recovery was enabled: a total of 178 were accepted in both settings, whereas 39 required REF/ALT exchange. The separate chain inspection placed 13 unavailable candidates in hg38-to-hg19 alignment gaps and one in a gap on the return path. These 14 remained unavailable for SGDP description; the chain gaps are not evidence of biological deletions. This agreement concerns coordinate and allele representation, not individual-genotype accuracy.

**Figure 4:**
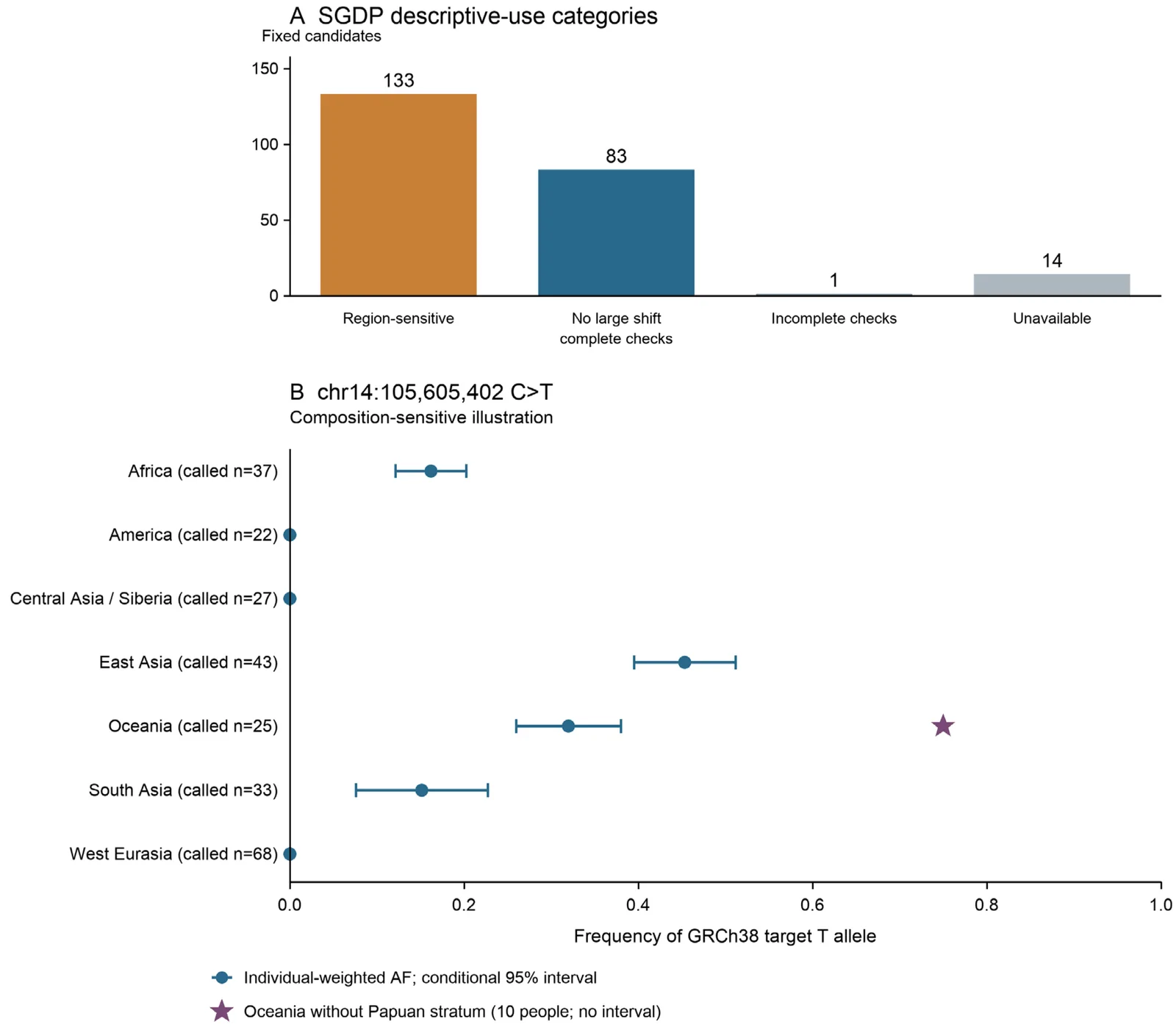
SGDP composition and conditional allele-frequency summaries. (**A**) All 231 fixed candidates by descriptive-use status, including 14 unavailable and one incompletely assessed candidate. No large shift within completed checks is not an accuracy classification. (**B**) Existing composition-sensitive example chr14:105,605,402 C>T, showing the GRCh38 target-T frequency under GP90_AD8, with the number of participants with available genotype calls and 95% within-Population-ID bootstrap intervals. The star marks the Oceania point estimate after excluding the Papuan stratum, with 10 participants remaining; it has no new confidence interval and is not a corrected frequency. Precise equal-Population-ID frequencies are omitted from the display to limit inference about very small strata; their aggregate sensitivity results are reported in Section 3.5. SGDP descriptions are not independent replication of 1KGP, and these conditional intervals omit imputation, technical and regional-composition uncertainty. For panel A, a region-sensitive designation requires an absolute frequency shift ≥0.05 in at least one supported quality, weighting or leave-one-Population-ID-out comparison. Each regional sample being compared must have ≥10 called individuals and a call rate ≥0.90; for population removal, both the full and remaining regional samples must meet these thresholds, and the removed population must contribute called genotypes. Equal-population weighting additionally requires calls from every included population. A candidate is flagged if any region meets this rule; insufficient support alone does not constitute sensitivity.

The geographic summaries were more sensitive to population composition. Equal-Population-ID weighting changed frequency by ≥0.05 in 92/1519 summaries. Across the declared checks, 133 candidates required region-specific sensitivity reporting, 83 had complete checks without a large shift, one had incomplete checks and 14 were unavailable (Figure 4A). Supported leave-one-population-out shifts ≥0.05 occurred for 15 candidates in Africa, 32 in America, six in Central Asia/Siberia and 96 in Oceania, with none in East Asia, South Asia or West Eurasia under these checks.

At chr14:105,605,402 C>T, the Oceania target-T frequency was 16/50 = 0.32 among 25 participants, with a fixed-Population-ID bootstrap interval of 0.26–0.38. Excluding the 15-person Papuan stratum gave 15/20 = 0.75 among the remaining 10 participants (Figure 4B). The latter is not a corrected frequency or a reason to remove participants. The example shows why population composition must accompany regional frequencies even when a within-stratum interval is narrow. SGDP broadens the geographic description but does not independently replicate 1KGP FST or remove reference-panel imputation dependence (Supplementary Data S9 and S10).

### 3.6. Measurement and Sampling Checks Bounded the Biological Interpretation

**Figure 5:**
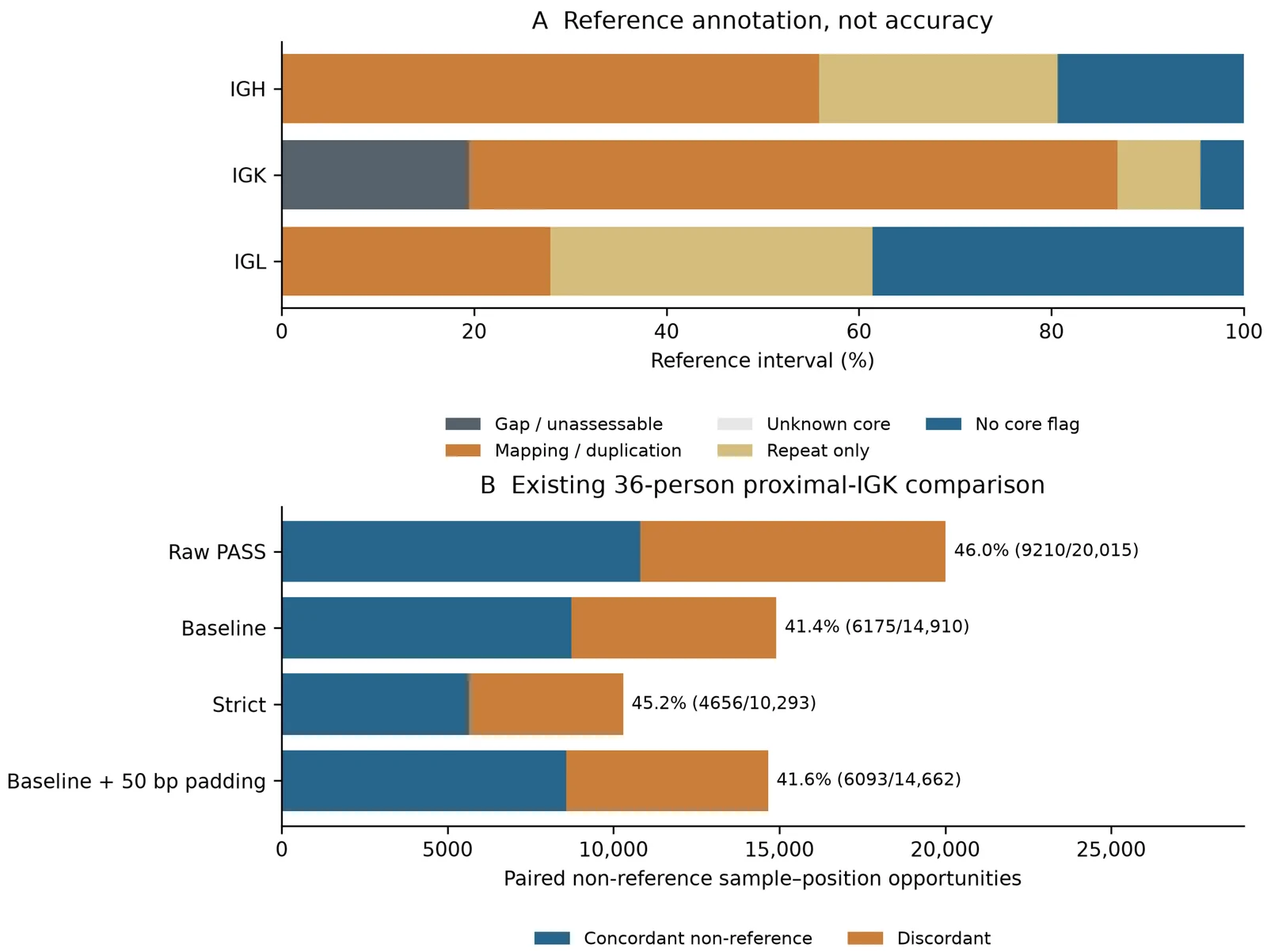
Reference-risk distribution and the cost of filtering. (**A**) Complete five-category 100-mer reference annotation across each locus; zero-width categories remain in the legend. Physical interval proportions are not genotype-error rates. (**B**) Concordant and discordant paired non-reference sample–position opportunities across four quality settings in the same 36-person proximal-IGK comparison. Labels give disagreement percentage and numerator/denominator at each setting. The comparison is local and copy assignment remains conditional; neither source is designated an unquestioned truth set. Changed denominators and concordant losses prevent interpreting a smaller discordant count as proof of improved accuracy.

The existing 36-person proximal-IGK comparison showed substantial disagreement and filtering losses (Figure 5B). Among paired non-reference sample–position opportunities, disagreement was 9210/20,015 (46.02%) in raw PASS calls, 6175/14,910 (41.42%) after baseline filtering and 4656/10,293 (45.23%) under strict filtering. Stricter filtering therefore did not monotonically reduce disagreement. In the assembly-first view, 1154 observations had VCF 0/0, 236 lacked a VCF record, and 78 had unusable VCF genotypes, alongside concordant, different non-reference and complex-context observations. These discrepancies could not be assigned exclusively to short-read errors. Excluding mapping/duplication flags from the baseline comparison removed 99.22% of discordant opportunities but also 90.12% of concordant non-reference opportunities, retaining only 6.11% of baseline non-reference opportunities. This local comparison does not validate the IGH/IGL candidates or establish true false-positive and false-negative rates (Supplementary Data S1 and S2).

Locus-level FST depended on site selection, especially at IGK. For AFR–EAS, primary Weir–Cockerham/Hudson estimates were 0.1386/0.1410 in IGH, 0.2303/0.2394 in IGK and 0.1231/0.1252 in IGL. The IGK Weir–Cockerham estimate fell to 0.0669 under strict genotype quality, 0.0504 at the 98% baseline call-rate threshold and 0.1150 after local LD pruning. Baseline-to-strict maximum differences across pairs were smaller on common estimable positions (IGH/IGK/IGL: 0.0008/0.0089/0.0005) than with separately selected positions (0.0511/0.1635/0.0061). Balanced sampling shifted median estimates by at most 0.0039/0.0137/0.0042. Primary 50 kb spatial intervals were available for all IGH and IGL pairs but unavailable for IGK, which had only six informative blocks; at 100 kb, none of the loci met the reporting requirement. These results do not support an intrinsic ranking of locus conservation or selection pressure (Figure 2A; Supplementary Data S3).

Candidate classification was also vulnerable to specified changes in accepted genotypes. In the convergent expected-count scenario, 53/425 candidate–pair comparisons lost the high classification at a 1% replacement-opportunity fraction: 15/132 in IGH and 38/293 in IGL. This is a hypothetical perturbation, not a measured sequencing-error rate or estimated false-discovery count. The complete eight-scenario curves include changes that increased differentiation (Figure S1; Supplementary Data S11). In the pre-filter risk-background analysis, mapping/duplication-flag contrasts under the primary baseline Weir–Cockerham setting were higher for high-FST sites in seven locus–pair comparisons, lower in 12 and equal in two; nine lacked shared support. Support covered only 6.8–25.8% of IGK high sites, limiting generalization (Figure S2; Supplementary Data S12).

The archived seven-group categories were also panel-dependent: only 1399/3024 IGH, 455/1031 IGK and 856/2543 IGL coordinates in the stored highest category retained that category in the 10-pair subpanel excluding Central Asia/Siberia and Oceania. More than half of the archived raw FST entries were unavailable at each locus, and duplicate-coordinate and label–number conflicts prevented complete reconciliation. These findings motivated the separate-cohort design and explicit candidate rule used here; they do not identify particular populations as sources of false positives (Supplementary Data S13).

In the regional Picard audit, 119,660 input records comprised 115,949 simple biallelic SNVs and 3711 indel/complex records. With swapped-reference recovery disabled, 3963/51,998 IGH records (7.62%), 328/30,285 IGK records (1.08%) and 1166/37,377 IGL records (3.12%) were rejected. Enabling recovery retained 2448 additional SNVs, reducing the respective rejected counts to 2385 (4.59%), 181 (0.60%) and 443 (1.19%). Of the 3009 remaining rejections, 2992 had no usable target under the specified chain and matching threshold, 12 had mismatched reference alleles and five were indels spanning multiple conversion intervals. Accepted records outside the frozen main IG intervals were retained and flagged: 7633 without recovery and 7713 with recovery, including 385 and 424 IGH-source records mapped to other chromosomes. Conversion counts therefore cannot be substituted for final analysis-site counts or sequencing-error rates. The complete per-record results are in the coordinate-audit deposit; they describe this new run, not the unavailable historical Picard execution.

## 4. Discussion

This study connects population-differentiated reference SNVs with specific expression and trait records relevant to antibody variation. The 231 conditional candidates provide a defined starting set, and their annotations identify molecular questions that a frequency comparison alone would not specify. Most candidates with GTEx matches were in IGL, while the GWAS overlaps predominantly concerned protein measurements and one seropositivity label. These traceable links between candidates and measurements are the main contribution of this study. Differentiation alone does not establish functional importance, and the quality, LD and sampling analyses set the limits of interpretation. Because the candidate rule was defined after inspection of aggregate sensitivity results, although before generating the candidate lists, the candidate set is exploratory and hypothesis-generating rather than the product of a prospectively defined analytical framework.

IGLV4-69 gives the rs2856876 observation a specific antibody-repertoire context. This light-chain V gene is used by experimentally characterized neutralizing antibodies, including the IGHV3-66/IGLV4-69 antibody YB12-197 against SARS-CoV-2 and three A35-binding antibodies against monkeypox virus [19,20]. In our data, a marked observed EAS–EUR allele-frequency difference coincided with a whole-blood IGLV4-69 expression association and a protein-level trait carrying the same gene label. A testable hypothesis is that inherited variation tagged by this candidate contributes to differences in the availability or recruitment of IGLV4-69-using B cells, influencing the composition of selected antigen-specific responses. The published activities belong to complete paired antibodies; the present SNV and its effect on repertoire usage have not been functionally established.

The RNA and protein measurements must also be distinguished. LD, cell composition or assay assignment could explain the overlap of IGLV4-69-labeled RNA and protein records. The original GWAS effect alleles and their alignment with the GTEx records remain unresolved; transcript assignment and protein-assay specificity also require verification. The spleen association concerns IGLC5, a pseudogene, and cannot support a functional constant-region protein mechanism. The candidate’s LD removal and SGDP composition sensitivity further preclude treating these records as an independently replicated molecular mechanism or evidence of stronger immunity in a particular population. The available evidence does not justify prioritizing rs2856876 over the other candidates; it is presented only to illustrate how population differentiation can be connected to existing molecular annotations.

IGLV8-61 provides a complementary clinical research context. A study of patients receiving the oncolytic adenovirus TILT-123 reported associations between IGLV8-61-containing natural IgM and clinical outcomes; recombinant IgM antibodies obtained from one responding patient enhanced viral transduction and tumor-cell killing in vitro [21]. Its occurrence among our expression targets and protein-level trait labels motivates asking whether differentiated IG variation is associated with the abundance or repertoire usage of antibody species involved in virus–antibody interactions.

Non-coding variation offers another route from inherited diversity to repertoire composition. Using long-read germline sequencing and matched antibody repertoires, Engelbrecht et al. associated upstream IGLV9-49 variation with gene usage and identified gene-usage QTLs involving IGLV8-61 and IGLV7-46 [3]. All three were expression targets in our annotation set. These findings provide a rationale for studying whether differentiated haplotypes affect how often particular light-chain segments enter the expressed repertoire. Our bulk-tissue eQTL overlaps measure a different phenotype and do not establish that the same variants underlie the published gene-usage associations.

Expression context also changes the interpretation of candidate counts. The IGHV1-69D records identify a regional association pattern across four bulk tissues, not 21 independently supported regulatory mechanisms in whole blood. The better-characterized IGHV1-69 example illustrates why this distinction matters: F/L allele families differ at a residue implicated in influenza antibody recognition, and genotype has been associated with usage of other IGHV genes [4]. Such allele- and locus-dependent observations cannot be transferred to IGHV1-69D by name similarity. Likewise, the source study behind the rs8009594 overlap measured antibody–peptide reactivity [18]; its catalog label is closer to a defined immune measurement than to a disease-risk outcome. Together, these examples suggest useful follow-up endpoints—gene-specific expression, repertoire usage and antibody reactivity. This is consistent with the original rationale for integrating population variation and functional resources while requiring a more specific interpretation of what each resource actually measured.

The molecular-trait overlaps also raise questions about how inherited differences interact with immune and protein homeostasis. Changes in IG gene sequence or availability can affect repertoire composition [2,3], but protein abundance alone does not establish altered folding, localization or turnover. A cross-disease analogy is provided by Huang et al., who review how TDP-43 protein pathology may interact with mitochondrial quality control and organelle communication to modify neuronal vulnerability in Alzheimer’s disease [22]. For IGLV4-69 and related candidates, this perspective motivates testing whether validated molecular differences have context-dependent effects on antibody production or cellular stress.

The background comparisons set a limit on this interpretation. Although 177 candidates matched significant GTEx catalogs, supported high-versus-low contrasts showed no consistent high-FST advantage. Our results therefore do not justify preferentially assigning regulatory function to the candidate set as a whole. However, the absence of a higher catalog-presence rate does not rule out a function for a particular allele. The comparison was restricted by shared local strata and significant-only input tables; it did not cover the full tested universe or correct for cell composition. Bulk-tissue IG expression may depend on B-cell and antibody-secreting-cell abundance, activation and clonal composition, while local LD and ambiguous transcript assignment can generate related associations [16]. A useful next experiment must distinguish these explanations rather than simply reproduce a catalog overlap. Future studies could combine verified donor genotypes with single-cell RNA sequencing to test eQTLs within defined B-cell subsets and activation states, helping distinguish within-cell-type expression associations from differences in cell abundance [23]. Paired B-cell receptor sequencing could further characterize clonal composition and IG gene usage. Such analyses require adequate numbers of independent donors and reliable transcript assignment; single-cell resolution alone would not establish a causal allelic effect.

Population context helps determine which alleles and haplotypes a follow-up study needs to sample. SGDP extends this description to additional geographic groups, but its observed regional frequencies can depend strongly on the populations from which participants were sampled. The Oceania example shows that narrow conditional intervals and large composition shifts can coexist. Keeping the cohorts separate and reporting these sensitivities preserves that geographic information without labeling it independent replication. The broad regional labels should not be treated as homogeneous biological groups or as proxies for individual disease susceptibility. Descriptive concordance would mean similar observed allele frequencies or differentiation patterns across datasets; even if observed, it would not establish genotype accuracy or biological function. Given imputation dependence and the lack of adequately matched population samples, we deliberately retained SGDP as a geographic description rather than performing a formal cross-cohort concordance analysis; biological validation requires orthogonal genomic confirmation and appropriate functional experiments.

Several constraints limit the biological claims. IGH and IGL candidates survived a specified threshold rule, not orthogonal validation; few belonged to the local LD-retained set, some conditional intervals crossed the threshold, and hypothetical perturbations changed classifications. IGK estimates were particularly sensitive to the selected sites, so zero retained IGK candidates cannot establish conservation. The proximal-IGK comparison was limited in region and copy assignment and did not estimate true sensitivity or specificity. Taken together, the mappability, duplication and local assembly-comparison analyses show that the reported candidates remain conditional on limitations of reference-based short-read genotyping in IG loci. Filtering does not establish correct paralog assignment or exclude individual structural variation and somatic rearrangement. Specialized IG genotyping and haplotype-resolved approaches [24,25,26] offer complementary follow-up strategies, rather than validation already provided by the present analyses.

The clinical motivation is to understand inherited differences that may contribute to antibody responses. Previous studies have demonstrated allele-dependent antibody activity and connections between genomic variation and repertoire composition using designs suited to those questions [2,3,4,5]. Here, a practical follow-up would first verify selected variant and haplotype identities in suitable genomic material, then test the particular molecular measurements suggested by the annotations. For the IGL example, this means distinguishing gene copies and transcripts, resolving the original protein assay and effect alleles, and assessing expression or repertoire usage with cell composition accounted for. Only subsequent functional and clinical evidence could connect such measurements to protection, autoimmunity or vaccine responsiveness. The resource helps specify these questions and the samples needed to investigate them.

After genomic confirmation, precise allele replacement in a suitable human B-cell model could generate isogenic lines differing at the candidate nucleotide, using base or prime editing as permitted by the substitution and local sequence [27,28]. Comparing independently edited clones and appropriate controls for gene-specific expression would help isolate allelic effects from linked variation. Editing outcomes and unintended changes would need verification; repertoire-level effects would require a model that captures B-cell development and rearrangement. These are proposed validation strategies, not experiments performed here.

## 5. Conclusions

This reference-based survey describes 231 conditional population-differentiated IG candidates and provides expression and trait annotations for the matched subsets, offering specific starting points for studies of antibody variation. The rs2856876 example connects an observed population-frequency difference with IGLV4-69-labeled RNA and protein associations that warrant examination at the variant, transcript and assay levels. SGDP adds geographic context with explicit composition sensitivity. The lack of consistently higher GTEx catalog-presence rates, together with unresolved measurement limitations, constrains general functional interpretation. These candidates are a resource for targeted biological follow-up.

## Figures and Tables

**Table 1:** Cohort roles and retained sample sizes.

Cohort	Geographic Label	People	Role
1KGP	AFR	649	Primary FST
1KGP	AMR	347	Primary FST
1KGP	EAS	504	Primary FST
1KGP	EUR	503	Primary FST
1KGP	SAS	485	Primary FST
SGDP	Africa	37	Dependent description
SGDP	America	22	Dependent description
SGDP	CentralAsiaSiberia	27	Dependent description
SGDP	EastAsia	43	Dependent description
SGDP	Oceania	25	Dependent description
SGDP	SouthAsia	33	Dependent description
SGDP	WestEurasia	68	Dependent description

The cohort totals are not added as an independent combined sample. Removal of shared IDs does not remove imputation dependence. Source strata and original population labels remain distinct.

**Table 2:** Reference-filter and primary-analysis site denominators.

Locus	S1 Sites	S2 Sites	S3 Sites	Primary Selected	Candidates
IGH	70,344	28,594	15,582	5843	49
IGK	56,134	10,864	3533	1273	0
IGL	43,044	29,299	17,141	12,276	182

Primary selected: S3/baseline/95% in all five groups, before pair-specific estimator eligibility. Candidates use both quality stages and both estimators plus the additional rules in Section 2.6. Counts are sites, not candidate–pair records; S1–S3 filtering is not validation.

**Table 3:** Complete coordinate-level GWAS overlaps among the 231 candidates.

Candidate Allele (GRCh38)	RefSNP	Original GWAS Trait Labels	N
IGH chr14:106,777,494 G>T	rs8009594	Seropositivity for ruminococcus torques (agilent_118810)	1
IGH chr14:106,877,229 A>G	rs7144412	CHAT protein levels	1
IGL chr22:22,182,108 A>G	rs6001213	IGLV8-61 protein levels	1
IGL chr22:22,194,092 T>C	rs1543779	IGLV6-57 protein levels	1
IGL chr22:22,202,639 T>C	rs2330031	IGLV8-61 protein levels; IGLV4-60 protein levels; SOCS5 protein levels	3
IGL chr22:22,211,276 C>G	rs6001394	Circulating IKZF2 levels	1
IGL chr22:22,907,270 C>A	rs2856876	Forkhead box protein J2 levels; ER membrane protein complex subunit 8 levels; serum levels of protein FOXJ2; serum levels of protein SCP2D1; IGLV5-45 protein levels; IGLV8-61 protein levels; IGLV4-69 protein levels; IGLV7-43 protein levels; MAB21L2 protein levels; IGLC2 protein levels	10

N counts association records, not independent signals or diseases. Trait labels are reproduced from the source catalog; identical labels within a coordinate are listed once. All seven candidates were removed by the inherited local LD procedure. RefSNP membership does not establish the original-study effect allele or risk direction. Full source records, including study identifiers and effect fields, remain in Supplementary Data S8.

## Data Availability

Public project resources are available from the 1000 Genomes Project (https://www.internationalgenome.org/ (accessed on 27 September 2026)), the Simons Genome Diversity Project (https://www.simonsfoundation.org/simons-genome-diversity-project/ (accessed on 27 September 2026)), the GWAS Catalog (https://www.ebi.ac.uk/gwas/ (accessed on 27 September 2026)) and GTEx (https://gtexportal.org/home/ (accessed on 27 September 2026)). Source versions, local file identities, unresolved lineage limitations and analysis parameters are documented in the accompanying Supplementary Materials. Both the coordinate-conversion audit and the expanded derived-data and code package are now publicly available in Zenodo at https://doi.org/10.5281/zenodo.22740793 [29].

## Supplementary Materials

The following supporting information can be downloaded at: https://www.mdpi.com/article/10.3390/genes17101216/s1. Supplementary Methods: source versions, analysis parameters, interval definitions, limitations and data dictionary; Figure S1: Complete deterministic genotype-replacement sensitivity scenarios; Figure S2: Full primary five-category reference-risk contrast before S2/S3 exclusions; Supplementary Data S1: Local proximal-IGK comparison; Supplementary Data S2: Reference-risk annotations and filtering losses; Supplementary Data S3: FST estimates and sampling sensitivities; Supplementary Data S4: Fixed candidates and selection accounting; Supplementary Data S5: Conditional candidate intervals; Supplementary Data S6: Exact-allele GTEx annotation; Supplementary Data S7: Support-limited GTEx background comparisons; Supplementary Data S8: GWAS coordinate overlaps; Supplementary Data S9: SGDP harmonization and public regional frequencies; Supplementary Data S10: SGDP descriptive-use categories; Supplementary Data S11: Deterministic genotype-perturbation scenarios; Supplementary Data S12: Five-category risk-background contrasts; Supplementary Data S13: Historical seven-group diagnostic. The journal supplement includes aggregate display data and plotting code for revised Figure 4, but not the complete underlying SGDP sensitivity outputs. Additional analysis code and historical source material are provided in the separately archived Genes_Reproducibility_i065.zip in Zenodo [29]. The separate coordinate-audit package contains sites-only regional inputs, accepted and rejected conversion outputs, record-level outcomes, candidate correspondence checks, redacted logs and coordinate-reproduction entry points. These public packages complement the journal supplement and omit individual-level and potentially disclosive fine-group outputs.
