# Supplementary material for: Study of Population-Differentiated Single-Nucleotide Variants in Human Immunoglobulin Loci Using Short-Read Sequencing Data
Source: Genes (Basel). 2026 Sep 30;17(10):1216. doi: 10.3390/genes17101216 (PMC13704469; doi:10.3390/genes17101216)
Supplement: Supplementary file 1 [file genes-17-01216-s001.zip › Supplementary_Methods and Figures.pdf]

Supplementary Methods and Data Guide

S1. Source identity, sample scope and coordinate accounting

SOURCE_MANIFEST.tsv in the journal data archive records the relative origin and SHA-256 checksum of each unchanged copied file. FILE_INDEX.tsv inventories the delivered files; TABLE_SCHEMA.tsv records the actual columns and row counts. Module-level input_manifest.tsv and reference_versions.tsv files retain available source identities and redacted historical paths. These records do not resolve the exact official parent release of the locally archived 1KGP and SGDP regional VCFs. Historical analysis code is separately available in code/historical within the published i065 archive at https://doi.org/10.5281/zenodo.22740793; references below to those analysis scripts refer to that archive, not to the journal archive's Figure 4 plotting script.

The primary cohort is the three-VCF-header intersection with the established 2,504-person 1KGP panel, followed by exclusion of 16 explicit pedigree redundancies. The corrected pedigree is integrated_call_samples_v3.20250704.ALL.ped; the population panel is integrated_call_samples_v3.20130502.ALL.panel. Known parent, sibling, child, second-order and third-order relationships and shared known parents create edges. Lexicographically sorted greedy retention acts on the induced panel graph. It is neither removal of all people sharing a family identifier nor a genome-wide kinship analysis. AFR/AMR/EAS/EUR/SAS retain 649/347/504/503/485 people. IDs outside the chosen panel are a scope exclusion, not evidence of bad data.

SGDP contains 255 of the 278 local identifiers after excluding 23 shared 1KGP IDs. The regional sample sizes are Africa 37, America 22, Central Asia/Siberia 27, East Asia 43, Oceania 25, South Asia 33 and West Eurasia 68. The seven regional labels are not equivalents of the five 1KGP strata. Six name-related cross-resource population groups contain only one to four SGDP individuals each and are not a validated ancestry-matched comparison set. Direct AF/FST concordance was therefore not presented. Source counts are 166 cell line, 74 blood and 15 saliva; those labels are not a causal LCL experiment.

Coordinates are GRCh38 one-based VCF positions unless an interval is explicitly labeled BED. Reference-risk BED ranges are zero-based and half-open. The local assembly diagnostic instead uses the closed, one-based interval chr2:88,880,000–89,260,000. A candidate key includes chromosome, position, REF and ALT. SGDP harmonization.tsv documents all 231 candidate outcomes, including target/source alleles, strand, forward/reciprocal hit counts, reference bases and recoding mode. Accepted reference/alternate swaps are not strand complements or position-only matches. The 14 unavailable candidates are retained in downstream usage accounting. The historical Picard full-region rejection ledger is unavailable, so its failure rate and per-locus cause distribution are not reconstructed from count subtraction.

A new forward-conversion audit was performed on the three retained local SGDP GRCh37 regional snapshots, independently of the historical execution. All 119,660 physical records were projected to sites-only VCFs without merging duplicates, and contigs 2/14/22 were explicitly renamed chr2/chr14/chr22. Picard 2.23.0 LiftoverVcf used the recorded hg19ToHg38 chain and the exact assembled GRCh38 target reference, LIFTOVER_MIN_MATCH=1.0, VALIDATION_STRINGENCY=STRICT and WARN_ON_MISSING_CONTIG=false. RECOVER_SWAPPED_REF_ALT=false was the baseline and true the sensitivity. Accepted/rejected membership was determined from output files rather than inherited FILTER values. Every source ID was accounted for once per mode; accepted records outside the original analysis interval were flagged. The separate 231-candidate reverse/reciprocal correspondence query has a different direction and denominator. The published i062 archive at https://doi.org/10.5281/zenodo.22740793 supplies new inputs, outputs, parameter and reference checksums, logs and replay scripts. It measures behavior of this

new audit, not the missing original run, and does not establish genotype accuracy or population-specific error rates.

S2. Reference categories, genotype filters and local comparison

For each mappability scale, the category precedence is: gap if a gap is present; mapping/duplication flag if a segmental duplication is present or a known multi-read mappability value is less than $1-10^{-6}$; UNKNOWN if a required core annotation is missing; repeat only if RepeatMasker or simple-repeat overlap remains; otherwise no core flag. Core completeness is recorded separately. A known flag can take precedence over another missing annotation; UNKNOWN is not equivalent to zero overlap. At 50-mer scale the corresponding mappability track replaces the 100-mer track. Segmental-duplication identity ≥0.98 is an auxiliary field, not the sole primary duplication criterion. DGV acquisition/identity information does not supply individual SV calls; DGV was not projected into a per-sample validation claim.

S1 is simple biallelic SNV plus literal FILTER=PASS. S2 is S1 plus complete 100-mer annotation and either repeat-only or no-core-flag status. S3 is S1 plus complete 100-mer annotation and no-core-flag status. S3 does not mean high confidence. Exact local record eligibility and complex-span exclusions are implemented in the historical i036/i037 scripts in the separately deposited i065 archive; complex or multiallelic records are not decomposed for candidate discovery.

The 1KGP status code preserves the first failure among missing/partial/non-diploid/invalid GT, missing/invalid/below-threshold quality, missing/invalid heterozygous AD and out-of-range allele balance. Valid non-reference and heterozygous flags are separate from the quality-stage status. Baseline requires GQ≥20, DP≥10 and, for heterozygotes, 0.20≤AD_ALT/(AD_REF+AD_ALT)≤0.80. Strict requires GQ≥30, DP≥15 and heterozygous balance 0.25–0.75. AD is not an extra balance requirement for homozygotes. The primary all-five-group call-rate threshold is 95%; 90% and 98% are sensitivities. Failed genotypes reduce the called denominator rather than becoming 0/0.

The local proximal-IGK comparison counts sample–position opportunities, not independent variants. A paired non-reference opportunity requires evaluability in both representations and a non-reference genotype in at least one. Reference/reference pairs are not its denominator. Assembly-first observations separately include absent VCF records, invalid GT and complex contexts. The resulting discrepancy rates must not be reported as specificity, sensitivity, precision or false-positive/false-negative rates. The original assembly naming/copy interpretation is conditional and ancillary sequence-context verification was partial. The 50-bp boundary-padding sensitivity has a different retained denominator. Filter loss of concordant non-reference opportunities is reported alongside discordance removal.

S3. FST, local LD and three different sampling summaries

Custom count-based code implements Weir–Cockerham and Hudson components. A locus estimate is the sum of numerators divided by the sum of denominators over jointly eligible sites. It is not the mean site FST. The exact component calculations are in the separately deposited historical i038_fst.py and the frozen fst.json in Supplementary Data S3. All-five-group sample support is required for a selected site; pair-specific estimator eligibility is then recorded. Joint same-allele fixation is non-estimable, not FST=0; opposite-allele fixation can be estimable. Signed finite estimates are retained. Historical categorical parsing and tiny signed floating-point values have separate semantics, so auxiliary negative-site counters should not be treated as precise biological counts near zero.

Local LD sensitivity uses a 50-kb ascending-position greedy procedure on globally polymorphic primary sites. Later sites are dropped if any broad group has an eligible pairwise-complete dosage $r^2>0.2$ with an already retained site. Eligibility requires ≥20 jointly called people, joint minor-allele count ≥5 and nonzero variance.

Many pair–group comparisons are unassessed. The output is a locally screened subset, not a set proven independent across the region or genome. PLINK was not used for this step.

Three uncertainty/sensitivity procedures must be distinguished. Spatial resampling draws chromosome-anchored 50-kb or 100-kb blocks with replacement, includes the declared locus geometry and uses 1,000 replicates (seed 20260908). A percentile interval needs ≥10 informative blocks and ≥950 valid draws. Primary 50-kb IGH and IGL intervals satisfy that rule; IGK does not. Primary 100-kb intervals do not satisfy the informative-block threshold for any locus. A missing interval is not a zero-width interval.

Balanced sampling draws 200 people per broad group without replacement, using proportional quotas for the 26 original population strata and largest-remainder allocation, across 100 replicates (seed 20260909). The full-sample primary mask is fixed; 95% call-rate selection is not reapplied after each draw. The draw distribution is not a 95% confidence interval. The supplement retains every pair's values, so readers can see which comparisons changed rather than relying only on locus-wise maxima.

Fixed-candidate individual bootstrap uses 2,000 within-original-population resamples with replacement (seed 2026090840), preserving stratum sizes and using the same weights across candidates and quality stages. Percentile limits require ≥1,900 valid draws and use linear quantiles. These are conditional on selected sites, observed genotypes and sample composition, not selection-adjusted, simultaneous or total-error intervals. All 1,700 FST rows and 2,310 AF rows retain valid-replicate and empirical-degeneracy fields. A degenerate empirical distribution is not proof of zero population uncertainty.

S4. Candidate rules and matching support

Candidate discovery begins after aggregate sensitivity inspection, not before the study. Eligibility requires S3 baseline95 AND S3 strict95; finite positive denominators for both estimators at both stages; per-stage maximum all-five-group call-rate spread ≤0.02; and pair-pooled minor-allele count ≥20 at both stages. The spread decision uses exact integer cross-products against 1/50. The ALT-frequency direction is also tested using integer cross-products. High requires all four FST values $>0.25+10^{-10}$ and matching nonzero ALT-difference direction; low requires all four $<0.05-10^{-10}$. Any value within the inclusive numerical guard of a threshold receives a boundary designation before high/low assignment.

Sensitivity cutoffs are 0.15 and 0.35; minor-allele-count requirements are compared at 0 and 20. No additional cutoff on the range of the four estimates defines quantitative stability. Unique candidates and candidate–pair records are different units. The inherited LD label refers to the earlier local pruning, not new pruning of the candidate set. The reported 231 candidates should not be called 231 independent signals.

IG-local annotation comparison strata use locus, pair, chromosome-anchored 100-kb block, baseline pair-pooled minor-allele-frequency bins [0,0.01,0.05,0.10,0.20,0.30,0.40,0.50], and minimum call-rate bins [0.95,0.98,1.0] across both stages and all groups. Bins are left-closed/right-open except the final inclusive upper endpoint. Shared-cell low rates are weighted by the number of high comparisons in each cell. The original five-controls-per-case capacity field does not mean that 1:5 matching was performed. Actual comparisons use their documented support and weights, with equal-cell and inherited LD-subset sensitivities. P values are not generated.

S5. Hypothetical perturbation and risk-background endpoints

The eight scenario codes are high_ref, low_alt, converge, high_alt, low_ref, diverge, both_ref and both_alt. High/low are defined separately for each fixed candidate–pair using baseline ALT frequency. At each opportunity e, the selected group's expected counts mix its accepted GT distribution with the chosen homozygote. $AC'=(1-e)AC+2ent$ and heterozygote count $H'=(1-e)H$, where $t=0$ for reference and $t=1$ for alternate, while called n remains fixed. No missing genotypes are invented. The original high rule is

reevaluated on these expected counts without candidate reselection. Expected changed-GT fraction depends on the starting homozygote counts and differs from e.

The grid is deterministic, 0–0.20 inclusive in 0.001 increments, with no random seed or Monte Carlo sampling. It does not estimate E(FST), power, true false discoveries or a globally consistent perturbed callset. Directions across overlapping pairs can be incompatible as one biological error process. Figure S1 presents every declared scenario, including those that increase rather than decrease differentiation.

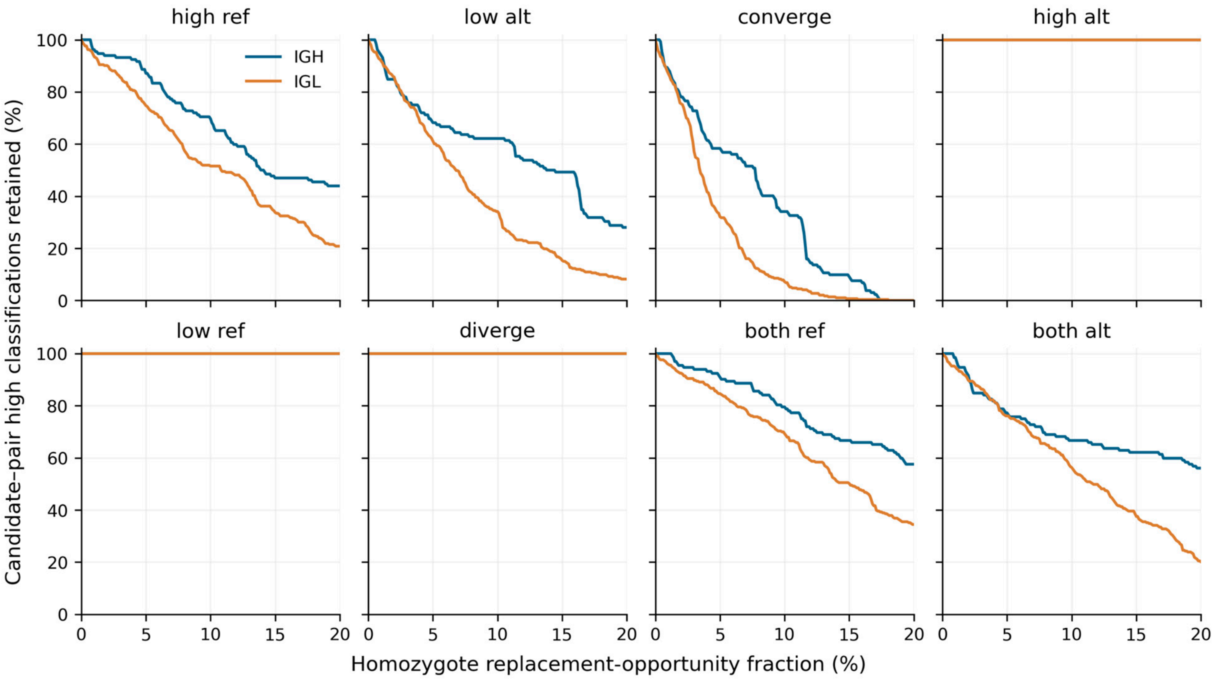

Figure S1. Complete deterministic genotype-replacement sensitivity scenarios. Every declared scenario is shown for IGH (132 candidate–pair comparisons) and IGL (293). The horizontal axis is homozygote replacement opportunity, not empirical error or actual changed-genotype fraction. The vertical axis is the fraction retaining the original four-estimate high rule. Values are FST evaluated at expected genotype counts, not expected stochastic FST or power. High/low orientation is fixed separately for each candidate–pair, so the scenarios are not one globally coherent modified callset. All original candidates remain in the denominator.

Reference-risk contrasts use pre-risk-filter S1 sites that meet the relevant stage's 95% all-group call threshold. Baseline WC84 is primary; baseline Hudson, strict WC84 and strict Hudson are separate settings. High/low thresholds use the numerical guard; matching uses the current setting's frequencies and call rates rather than transplanting the fixed-candidate background. The complete five-category 100-mer distribution is primary, with 50-mer sensitivity. A category contrast is a supported high fraction minus its high-weight-standardized low fraction, reported in percentage points. No support is NA, not a zero difference. The figure retains all 30 locus–pair units and supported/all-high counts, including unknown/gap categories even if their observed fraction is zero.

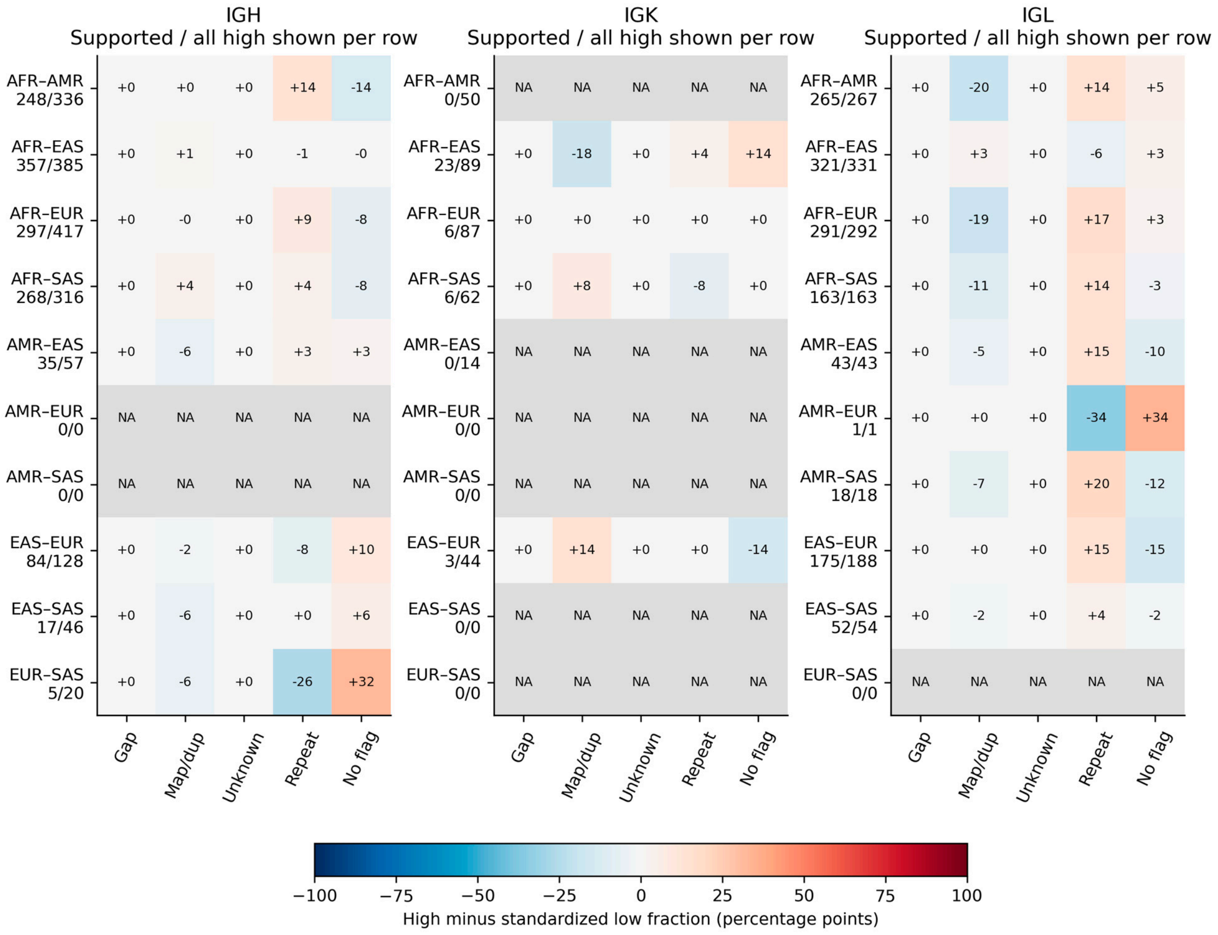

Figure S2. Full primary five-category reference-risk contrast before S2/S3 exclusions. Each row is one baseline-Weir–Cockerham S1/95% locus–pair comparison. Cells show high minus standardized-low fractions in percentage points; zero, negative values and unsupported gray NA remain distinct. Row labels give supported/all high-site counts. High and low are defined within this single estimator/stage setting, not by the four-estimate fixed-candidate rule. All five reference categories are retained. Lack of support, particularly at IGK, prevents extrapolation to all high sites; these descriptive contrasts are not enrichment tests. The unrounded values, 50-mer sensitivity and other estimator/stage settings are in Data S12.

S6. SGDP quality, intervals and composition

The raw scenario requires a usable diploid GT. GP90 additionally requires a valid GP vector with called-genotype probability ≥0.90 (sum tolerance 0.002); GP90_AD8 also requires AD total≥8 and each called allele supported by ≥2 reads. Quality filters do not make these imputed calls independent of their reference panel. PL is retained/recoded for identity consistency but is not the quality selection. The exact and swap modes preserve the GRCh38 target ALT definition. Failed mapping or required data are NA.

Local SGDP calculations distinguished genotype counts, AC/AN, expected and called participants, source and population levels, quality scenarios and interval status. The released public_regional_gp90_ad8_frequencies.tsv in Data S9 contains only the regional, source=all, GP90_AD8 projection, with expected_n and called_n both at least 10. A frequency uses its called chromosome denominator, not twice the complete source cohort size. Conditional intervals require called n≥10, call fraction≥0.90 and ≥950 valid draws among 1,000 within-Population-ID resamples (seed 20260909). Fixed stratum sizes mean that these intervals omit regional composition uncertainty. Full genotype-category, fine-

group, nested quality/source and exact equal-Population-ID frequency tables are not released. This disclosure threshold is not a certification of anonymity or complete redistribution rights.

Equal-Population-ID weighting gives each observed, called population stratum equal weight rather than weighting by participant count. Leave-one-Population-ID-out summaries recalculate the descriptive frequency on the remaining sample, with support conditions recorded for every removal. The ≥0.05 flag is a declared descriptive threshold. It does not identify an erroneous population or justify deleting a group to recover a preferred result. The chr14:105605402 example was selected as the already identified composition-sensitive illustration, not as an independently validated or clinically relevant allele. Its Papuan-removal point has no newly calculated confidence interval and is not plotted as an uncertainty limit.

Data S10 releases candidate-wide and candidate–region descriptive-use categories, not the complete numerical leave-one-Population-ID-out or equal-Population-ID tables. Revised Figure 4A has four status categories covering all 231 candidates. Figure 4B retains seven participant-weighted regional GP90_AD8 points and the existing Oceania Papuan-removed point. All eight display points, including the unavailable interval for the latter, are provided in Figure_4B_data.tsv. Precise equal-Population-ID points and the exact maximum-shift clue remain excluded. code/plot_figure4.py reproduces the display from the two Figure 4 tables; it does not recompute the underlying SGDP frequencies, intervals or composition sensitivities.

S7. Annotation identity, significance and historical status

GTEx input is the frozen v11 significant-cis-eQTL resource, including the four declared tissues. The source object was dated 15 January 2026 with generation identifier 1768503987699261. Matching requires exact b38 alleles. A candidate may have many correlated variant–gene–tissue records; unique sites, genes and gene–tissue pairs must be counted separately. Slopes, nominal P values and gene q values belong to the original source analysis, not the present population contrast. Source-listed lead status is not fine-mapping. Absence from the significant-only input is not proof that the variant was tested or lacked an effect. Bulk cell composition, transcript-assignment ambiguity and LD remain unresolved.

GWAS inputs use the 4 September 2026 full association download. Only single-coordinate records enter coordinate matching. The Supplementary Data S8 table retains all 18 hits and original traits, study accessions, P values and alleles. Exact RefSNP GRCh38 primary-sequence membership is a separate audit: it does not identify the allele used in the original study, particularly at multiallelic sites. No cross-study risk direction, disease-risk gradient, colocalization or causal mechanism is assigned.

The original seven-group diagnostic preserves all raw archived record denominators in all_archived_pair_status.tsv. Pair/position joins separately isolate the duplicate IGH coordinate; retained-category denominators are therefore unique evaluable coordinates rather than raw file rows. The stored-E analysis and numerical >0.25 / ≥0.25 analyses are separately named. The presence of high values only among CAS/OCE-involving pairs reflects panel dependence, not established small-sample artifacts. Raw NA means unavailable in the archive; its biological or technical cause is not inferred solely from the token. Unresolved label–number conflicts remain unresolved, and no successful subanalysis removes that source limitation.

S8. Package contents, code and reproducibility boundaries

Data	Source	Contents / distribution scope
S1	i035	Local proximal-IGK comparison (partial release)
S2	i036	Reference-risk annotations and filtering losses
S3	i038	FST estimates and sampling sensitivities (partial release)
S4	i039	Fixed candidates and selection accounting
S5	i040	Conditional candidate intervals (partial release)
S6	i041	Exact-allele GTEx annotation
S7	i043	Support-limited GTEx background comparisons
S8	i044	GWAS coordinate overlaps
S9	i046	SGDP harmonization and public regional frequencies (partial release)
S10	i047	SGDP descriptive-use categories (partial release)
S11	i049	Deterministic genotype-perturbation scenarios
S12	i050	Five-category risk-background contrasts
S13	i053	Historical seven-group diagnostic

The numbered collections are directories of related files, not thirteen individual worksheets. DATASET_INDEX.tsv and TABLE_GUIDE.md provide collection and file descriptions, units and interpretation limits. TABLE_SCHEMA.tsv lists every copied TSV column in its original order with the observed row count; source-specific dictionaries remain with S4, S5 and S11. FILE_INDEX.tsv, SOURCE_MANIFEST.tsv and checksums.sha256 support file identification and integrity checks. Numerical tables and the revised Figure 4/S1/S2 image files are byte-identical copies of the previously assembled distributable i067 material. Main-figure captions are synchronized to the author-selected manuscript. NA and status values preserve their source meanings and are not converted to zero. Historical execution and provenance files document their original analysis stage; embedded project-relative placeholders are not current public retrieval paths.

There is no Data S14 collection in this journal archive. The historical code formerly grouped as S14 is provided separately in code/historical within Genes_Reproducibility_i065.zip at https://doi.org/10.5281/zenodo.22740793. The same deposited package contains six eight-column sites-only filtered VCFs, masks, aggregate intermediates, configurations and available logs. These are site projections, not individual-genotype VCFs. The frozen primary analysis used Python 3.12.14 and NumPy 2.3.5, with Apple clang 21.0.0 recorded for native LD; per-module software tables specify other steps. The journal archive contains the standalone Figure 4 display script and its input tables. This script uses Python and Pillow plus an Arial or DejaVu Sans font; it requires a new output path and performs no genotype analysis.

The journal attachment and the deposited archives have complementary, partial reproduction coverage. The i065 aggregate replay entry point reproduces 540 FST summaries from packaged counts; it does not repeat all raw-genotype filtering, LD, individual resampling or source-dependent analyses. Original VCF genotype arrays, per-person comparison files, fine-group/nested SGDP frequency outputs and complete SGDP composition tables remain excluded. Exact upstream release identity and applicable individual-data redistribution terms remain unresolved, while small or differencing summaries can expose participant-level information. These are reasons for a restricted release, not a claim that all project-data reuse is prohibited. The coordinate audit supplies only the new run's logs; the original manuscript-era Picard logs were not recoverable. Revised Figure 4 display coverage is complete in this journal attachment, whereas the underlying

SGDP sensitivity pipeline remains only partially represented. The earlier i065 deposit is not claimed to contain this revised Figure 4 display bundle.

These materials support inspection of the reported calculations within the stated scope. They do not establish complete upstream provenance, orthogonal validation of the IGH/IGL candidate genotypes, a controlled LCL comparison, selection significance, matched non-IG enrichment, cell deconvolution or colocalization. The resource is intended for conditional candidate interpretation, not clinical risk prediction.
